# Anticancer activity, phytochemical investigation and molecular docking insights of *Citrullus colocynthis* (L.) fruits

**DOI:** 10.1038/s41598-023-46867-6

**Published:** 2023-11-16

**Authors:** Yasmine M. Mandour, Esraa Refaat, Heba D. Hassanein

**Affiliations:** 1 School of Life and Medical Sciences, University of Hertfordshire Hosted by Global Academic Foundation, New Administrative Capital, Cairo, 11578 Egypt; 2 Pharmacognosy Department, Pharmaceutical and Drug Industries Research Institute, National Research Centre, Dokki, Giza, Egypt; 3 Chemistry of Medicinal Plant Department, Pharmaceutical and Drug Industries Research Institute, National Research Centre, Dokki, Giza, Egypt

**Keywords:** Biochemistry, Plant sciences

## Abstract

Cancer disease is regarded as one of the most significant public health issues, regardless of economic standards. Medicinal plants are now regarded as a natural source of anticancer medicines due to their antioxidant and anti-mutagenic actions. Cucurbitaceae is considered to be one of the most economically significant families. One family species is *Citrullus colocynthis* (L.), which has a high concentration of many active secondary chemical metabolites. Various *C. colocynthis* plant extracts showed cytotoxicity against some cancer cells. This study aims to identify the *C. colocynthis* fruit components and determine whether they have anticancer action against MIA PaCa-2 and A431 cells. High-Performance Liquid Chromatography/Quadrupole Time of Flight/Mass Spectrometry (HPLC/QTOF/MS); the technique was accustomed to investigate the compounds of the ethyl acetate (EtOAc) fruit extract. Anticancer activity was investigated on both MIAPaCa-2 and A-431 cell lines. DPPH assay for antioxidant activity was carried out. Molecular modelling was employed to help understand the molecular basis for the observed anticancer activity. 24 compounds were tentatively identified by comparing the extract’s fragmentation pattern in positive mode against reference compounds spectra and literature. The EtOAc extract of *C. colocynthis* had effective positive results on cancer cells (MIAPaCa-2 and A-431) and was characterized by slight or no harmful effect on normal (healthy) cells. For the DPPH assay, EtOAc and BuOH extracts exhibited high antioxidant activity (86 and 76%, respectively) compared with the oxidative potential of the standard compound (Caffeic acid, 98%). One of the major cucurbitacin derivatives that LC/MS tentatively identified in the EtOAc extract was Cucurbita-5(10),6,23-triene-3β,25-diol. During this study, docking experiments and MD simulations were carried out, which suggested the anti-pancreatic cancer activity of *C. colocynthis* extract to be attributed to EGFR inhibition by Cucurbita-5(10),6,23-triene-3β,25-diol. Therefore, expansion of this type of research should be encouraged in the hope of obtaining natural therapeutics for cancerous tumors in the future, having the advantage of being cheaper, safer, and with fewer side effects.

## Introduction

Cancer is perceived as one of the top leading causes of death worldwide^1,2^. It is a heterogenous disease characterized by aberrant cell proliferation and invasiveness of abnormal cells which spread to neighboring tissues^3^. Medicinal plants have been widely used as a source of natural compounds for the treatment of cancer^4^. Besides their antitumor activity, natural compounds have many advantages owing to their low side effects, low cost, and ease of availability^5,6^.

The Cucurbitaceae family is considered one of the most economically significant families. It has 122 genera and 940 species, all found throughout the world's tropical and subtropical regions^7^. Due to their beneficial effects as dietary and therapeutic agents, many members of the Cucurbitaceae family are making significant contributions as domesticated species^8^. Most plants in this family are tolerant of dry seasons but intolerant of wet, frost-sensitive, and poorly drained soils^9^.

*Citrullus colocynthis* (L.) is a plant belonging to the family Cucurbitaceae. Its synonyms are *Cucumis colocynthis* and *Colocynthis vulgaris.* Other common names for this plant included in Cambridge English dictionary are bitter apple, and bitter gourd, while in Arabic it is known as Hanzal and Handal^6,10^. Mazher et al*.*^4^ previously reported *C. colocynthis* to have a high concentration of phenolics, flavonoids, glycosides, fatty acids, tocopherol, alkaloids, volatile chemicals, proteins, and amino acids.

*C. colocynthis* has been eaten and processed for its medical uses. It was traditionally used as a remedy mainly for diabetes mellitus along with a variety of diseases including gastrointestinal, musculoskeletal, neurological, cardiovascular, and respiratory disorders^4^. Their fruit extracts are astringent and treat jaundice, tumors, asthma, and urinary tract diseases. A study by Hussain et al.^8^ showed that fruit rind can treat bronchitis, arthritis, TB, and constipation. Additionally, the primary active ingredient in the fruits of *C. colocynthis*, colocynthin or cucurbitacin E-2-O-glucoside, has cathartic, antihistaminic, anticholinergic, negative chronotropic, and negative inotropic properties^7^.

Various *C. colocynthis* plant extracts showed cytotoxicity against some cancer cells^11–14^. However, very limited data are available on the activity of *C.colocynthis* fruit extracts on human pancreatic cancer cell lines (MIAPaca-2) and human skin cancer cells (A-431). Accordingly, the aim of this study is to evaluate the effect of *C.colocynthis* on pancreatic and skin cancer cells. The HPLC/QTOF/MS/MS technique to identify the possible compounds in the extract along with molecular docking and MD simulations to find the primary compound responsible for theobserved biological activities.

## Materials and methods

In this study, all methods followed the relevant guidelines/ regulations/ legislation.

### Preparation of plant extract

Around June 2022, the *C. colocynthis* (L.) fruits were purchased from a local Egyptian market. After the plant material was dried in an oven at 40 °C for five days, it was ground into powder using an electric mill. Until needed, the powdered sample was stored in an airtight container. A soxhlet extracted about 450 g of the powdered dry fruits over 72 h using solvents (2.5 L each) with increasing polarity. The plant extracts were concentrated in a rotary evaporator at 40–60 °C, and the finished extracts were stored in the fridge. Hexane (3 g), dichloromethane (8 g), ethyl acetate (13 g), butanol (11 g), and aqueous layer (21 g) were the five fractions that were obtained.

### HPLC–ESI–QTOF–MS/MS

HPLC–ESI–QTOF–MS/MS technique is used to look into the secondary metabolites of *C. colocynthis* fruit ethyl acetate extract. 50 mg of extract was dissolved in 1 mL of mobile phase A (water 0.1% formic acid), vortexed for 2 min, ultrasonically disrupted for 10 min, and centrifuged for 5 min at 10,000 rpm. The gas temperature and drying gas flow were 200 OC and 8 l/min, respectively. The injection had a 3 g/l concentration. The multi-step linear gradient was applied to mobile phase A (water 0.1%formic acid) at a flow rate of 3 ml/min for 45 min, starting at (90–10) percent of mobile phase B (Acetonitrile 0.1%formic acid). Columns: Agilent Technologies pre-Zorbax RP-18 column (dimensions: 150 mm 3 mm, dp = 2.7 m). For separation, a column at a temperature of 200 °C was utilized. The instrument used for chromatographic separation at the Faculty of Pharmacy- Fayoum University was the 6530 Q-TOF LC/MS (Agilent Technologies) equipped with an autosampler (G7129A), a quaternary pump (G7104C), and a column comp (G7116A). With a capillary voltage of 4500 V, ESI's ( +) ionization mode was used to acquire mass spectra. The mass spectra were captured between 50 and 3000 *m/z*. The collision energy was set to 10 V, while the skimmer and fragmentation voltages were set to 65 and 130 V, respectively.

###  Biological activity

#### Cytotoxicity

##### Cell culture

Human pancreatic cancer cell lines (MIAPaCa-2) and human skin cancer cells (A-431) were obtained from the American Type Culture Collection (ATCC). The cells were then cultured in T-25 flasks (Jet Biofil Flask) using 4–6 mL of DMEM-high glucose medium containing 10% fetal bovine serum (Gibson, 26140-079) and 1% antibiotic of penicillin–streptomycin (Gibson, 15140-122). They were raised at 37 °C in a 5% CO_2_ environment. The subculture's cells were washed twice with 1 ml PBS after removing the covering media when they were 80% confluence. The cells were pre-incubated for 1–2 min with trypsin–EDTA 0.25% (Gibson, 25200-056), and then the trypsin effect was countered by adding 3 ml of DMEM medium. They were put into 15 ml Falcon tubes and centrifuged at 1700 rpm for 7 min. The cells were pelleted, the supernatant medium was removed, the fresh medium was added, and the cells were placed in incubation flasks. 1 ml of freezing media (5–10% DMSO with 90–95% FBS) was used for freezing.

##### Using the MTT assay, determine cell toxicity

The colorimetric [3-(4, 5-dimethylthiazol-2yl-)-2,5-diphenyl tetrazolium bromide] tetrazolium reduction assay (MTT) was used to assess the vitality of the cells. A 96-well plate was used to seed the MIAPaCa-2 and A-431 cell lines, and 48 h of incubation were required. After incubation, the cells were treated to a final concentration of 100 ppm of the tested medicines in triplicates. 180 µl of the new medium was introduced to each well after the existing medium had been removed after 48 h. The MTT assay described by Mosmann ^15^ evaluated cytotoxicity. After treatment, the medium is removed from each well, and 20 µl of MTT (Sigma, Germany) is added. Each well is then incubated for 4 h at 37 °C in the dark. Following incubation, 200 µl of DMSO was added in place of the MTT solution. The cells were then placed in a shaking incubator for 10 min. An ELISA plate reader measured absorbance at 570 nm after adding glycine buffer.

##### Determination of IC_50_ values

Several concentrations of highly active samples that exhibited 60% cytotoxicity on various cancer cell lines were generated for dose–response investigations. The findings were utilized to determine the IC_50_ values of each sample in comparison with doxorubicin, a positive control.

#### DPPH assay

In the wells of a 96-well plate, 20 µl of extract that had been suitably diluted in DMSO was combined with 180 µl of DPPH in methanol (4 mg/ml) for the DPPH (2,2-diphenyl-1-picrylhydrazyl) radical scavenging experiment. After 15 min of darkness, the plate was placed in a Multiskan automatic kinetic microplate reader (Labsystems Multiskan RC reader) to measure the solution's absorbance at 540 nm. Standard (Trolox solutions in DMSO) and appropriate blanks (DMSO) were run simultaneously. The EC_50_ (the concentration that reduces DPPH absorbance by 50%) was determined by testing the extracts at different concentrations. This approach closely resembles that of earlier researchers^16^. Testing was done in triplicate.

$$\mathrm{RSA\%}= \frac{{Blank}_{(abs)}- {Sample}_{(abs)}}{{Blank}_{(abs)}} \times 100$$

### Molecular docking

Based on the literature, EGFR was selected as a drug target for pancreatic cancer^17^. The X-ray crystal structure of EGFR and its co-crystallized ligand (PDB ID: 5X2A)^18^ was obtained from the Protein Data Bank. The protein and the 3D structure of Cucurbita-5(10),6,23-triene-3β,25-diol were prepared using the structure preparation wizard in MOE (version 2019.01)^19^, and docking was performed using GOLD (version 5.8)^20,21^ using the ChemPLP scoring function as previously described^22,23^. PyMol was used for the generation of all figures^24^.

### Molecular dynamics

A 100 ns MD simulations was carried out using the PMEMD.cuda code of the AMBER Molecular Dynamics package^25^ for EGFR-Cucurbita-5(10),6,23-triene-3β,25-diol complex following the same previously described protocol of minimization, heating, density equilibration and production^26^. The trajectories were analyzed using CPPTraj^27^. Plots and visual inspection of the trajectories were done using XMgrace^28^ and VMD^29^, respectively.

### Ethics approval and consent to participate

The study was commenced after ethical clearance was secured from the National Research Centre Ethical Committee, Egypt, with protocol number (6441112202).

## Results

### Viability assay by MTT

**Figure 1:**
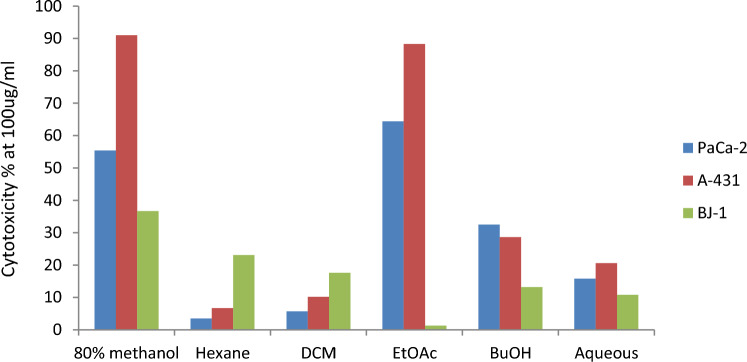
In vitro cytotoxic activity % (mean ± SD) of *C.colocynthis* extracts against pancreatic (PaCa-2) and skin (A-431) cancer cells, 48-h post-treatment as compared with (BJ-1) a normal cell line.

The viability of the MIAPaCa-2 and A-431 cell lines was evaluated using the MTT test after treatment with 100 µg/ml of *C. colocynthis* extracts. As seen in Fig. 1, the EtOAc fraction showed the highest cytotoxic effect, inhibiting PaCa-2 and A-431 by 54.4 and 68.3%, respectively. On the other hand, the extract showed only 1.3% inhibition on normal cells (BJ-1), indicating the extract to be selectively cytotoxic to cancer cells and not normal cells.

Additionally, the EtOAc extract demonstrated an IC_50_ value of 17.4 0.12 and 13.1 0.21 µg/ml of PaCa-2 and A-431 cancer cells, respectively. The cytotoxicity is nearly 2.3 and 1.3 folds more potent than the IC_50_ value obtained with the positive control drug, doxorubicin (21 1.2 and 37.6 1.5 µg/ml, respectively) (Table 1).

**Table 1:** In vitro cytotoxic activity (IC_50_ µg/ml) of *C. colocynthis* EtOAc extract and doxorubicin as a positive control against pancreatic (PaCa-2) and skin (A-431) cancer cells, 48-h post-treatment.

IC _50_ µg/ml		Cell line
EtOAc extract	Doxorubicin	
12.4 ± 0.12	28.3 ± 1.2	PaCa-2
19.1 ± 0.21	24.9 ± 1.5	A-431

### DPPH test

**Figure 2:**
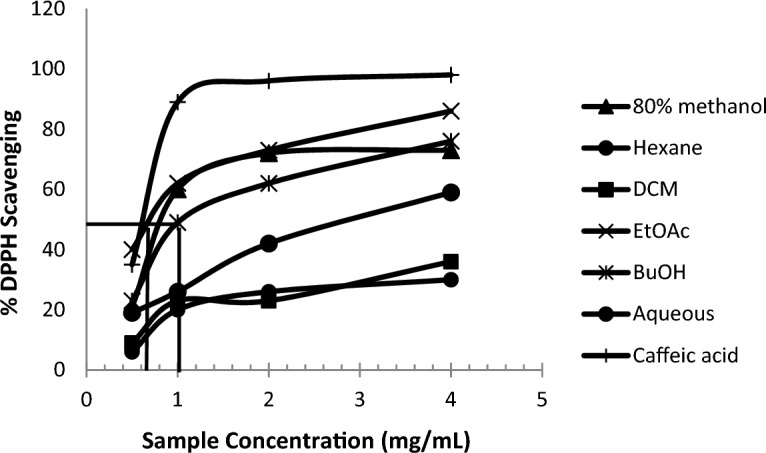
Concentration–response curves for the DPPH radical scavenging activity of Caffeic acid (positive control), different fractions of *C.colocynthis.*

Comparing the oxidative potential of the reference chemical (Caffeic acid, 98%) utilized in this study to the antioxidant activity of the EtOAc and BuOH extracts revealed their strong antioxidant activity in the DPPH experiment. The antioxidative activity of the EtOAc and BuOH extracts was significantly high (86% and 76%, respectively) with relative EC_50_ values of 1.0 and 0.62 mg/ml, respectively (Fig. 2).

### HPLC/ESI/MS/MS annotations of the main compounds of *C.colocynthis* ethyl acetate fruit extract

**Figure 3:**
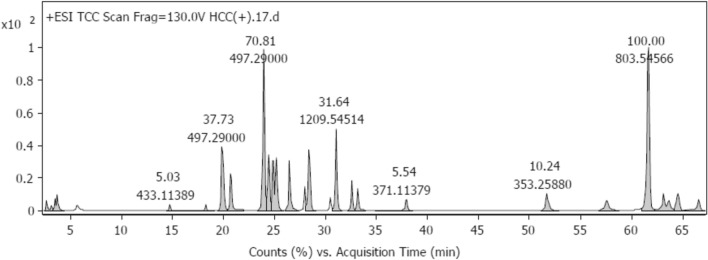
HPLC/MS/MS base peak chromatogram of *C.colocynthis* fruits in positive ionization mode.

In the current study, the chemical composition of the EtOAc extract was analyzed utilizing the positive ionization mode of the LC–ESI–QTOF–MS/MS method. By comparing the fragmentation pattern in the positive mode (Fig. 3) against the spctra of the reference compounds and literature, a total of 24 molecules shown in Table 2 were tentatively identified. The extraction solvent greatly impacts the chemistry of the materials under study. The limit of detection for each peak of chemicals was calculated using the Rt and whole MS spectra.

**Table 2:** Metabolites tentatively identified from *C.colocynthis* (L.) fruits ethyl acetate extract via positive mode HPLC-ESI-QTOF-MS/MS.

No.	Tentative identification	Molecular formula	Rt	Ionization ESI(+ /−)	Molecular weight	Theoretical (*m/z*) [M − H]^+^	Observed (*m/z*)	MS/MS production
1	Ferulic acid^30^	C_10_H_10_O_4_	0.11	[M − H]^+^	194.057	195.065	194.9014	145.0324, 149.0614, 177.0544, 195.065
2	Isovaleric acid^8^	C_5_H_10_O_2_	0.2	[M − H]^+^	102.068	103.076	103.937	58.0646, 61.0393, 103.076
3	1,4-Dideoxy-1,4-imino-d arabinitol^8^	C_5_H_11_NO_3_	0.7	[M − H]^+^	133.073	134.081	134.942	58.968, 84.962, 16.932
4	4-Hydroxy-l-threonine^8^	C_4_H_9_NO_4_	0.8	[M − H]^+^	135.053	136.061	136.22	58.968, 71.242, 123.43
5	Lysine^31^	C_6_H_14_N_2_O_2_	1.86	[M − H]^+^	146.1055	147.9543	147.1133	84.9573, 96.9986, 125.9832
6	Syringic acid^30^	C_9_H_10_O_5_	2.74	[M − H]^+^	198.893	199.06	198.893	83.983, 138.05, 166.961, 182
7	Chrysin^30^	C_15_H_10_O_4_	3.15	[M − H]^+^	254.058	255.066	255.971	143.0493, 209.0600, 253.0493
8	l-Carnitine^8^	C_7_H_16_NO_3_	4.35	[M − H]^+^	162.113	163.121	162.937	58.968, 84.957, 115.932
9	Cinnamic acid^32^	C_9_H_8_O_2_	5.14	[M − H]^+^	148.052	149.06	149.035	56.964, 84.957, 125.983
10	Vanillic acid^30^	C_8_H_8_O_4_	7.06	[M − H]^+^	168.04	169.05	169.08	64.017, 127.07, 143.99
11	Protocatechuic acid 4-glucoside^30^	C_13_H_16_O_9_	8.38	[M − H]^+^	316.079	317.087	317.68	61.01, 81.52, 113.0
12	Ascorbic acid^30^	C_6_H_8_O_6_	8.77	[M − H]^+^	176.032	177.04	177.075	64.02, 84.96, 134.96
13	Pantothenic acid^8^	C_9_H_17_NO_5_	10.2	[M − H]^+^	219.11	220.12	220.959	66.02, 110.01, 134.99
14	Apigenin^30^	C_15_H_10_O_5_	11.51	[M − H]^+^	270.05	271.06	271.86	55.98, 70.01, 116.93
15	Caffeic acid^32^	C_9_H_8_O_4_	11.93	[M − H]^+^	180.042	181.05	182	84.96, 123.03, 148.07
16	Lariciresinol-glucopyranoside^33^	C_26_H_34_O_11_	12.52	[M − H]^+^	522.202	523.21	523.981	82.026, 138.008, 186.99
17	Palmitic acid^8,31^	C_16_H_32_O_2_	13.55	[M − H]^+^	256.24	257.25	257.08	60.043, 143.99, 184.92
18	Cucurbita-5(10),6,23-triene-3β,25-diol ^34^	C_30_H_48_O_2_	16.14	[M − H]^+^	440.36	441.37	442.13	70.01, 299.80, 392.86
19	Apigenin-7-rutinoside^33^	C_27_H_30_O_14_	17.16	[M − H]^+^	578.155	579.16	579.28	55.05, 176.99, 318.89
20	Kaempferol 3-*O*-(6”-*O*-acetyl) glycoside ^33^	C_23_H_22_O_12_	24.41	[M − H]^+^	490.103	491.111	491.1615	101.05, 181.06, 245.09
21	Myricetin-3-*O*-glucoside^30^	C_21_H_20_O_13_	26.17	[M − H]^+^	480.090	481.098	481.3263	84.96, 143.99, 239.09, 317.179
22	Chicoric acid^30^	C_22_H_18_O_12_	27.05	[M − H]^+^	474.079	475.087	475.305	445.18, 453.20, 473.20
23	*m*-Coumaric acid^33^	C_9_H_8_O_3_	34.59	[M − H]^+^	164.047	165.055	165.0688	56.99, 84.96, 109.06

Data acquisition and analysis were performed using Agilent LC–ESI–QTOF/MS Mass Hunter workstation software (Qualitative Analysis, version B.06.00, Agilent 2012) and Mass Bank Data Base.

### Molecular docking

**Figure 4:**
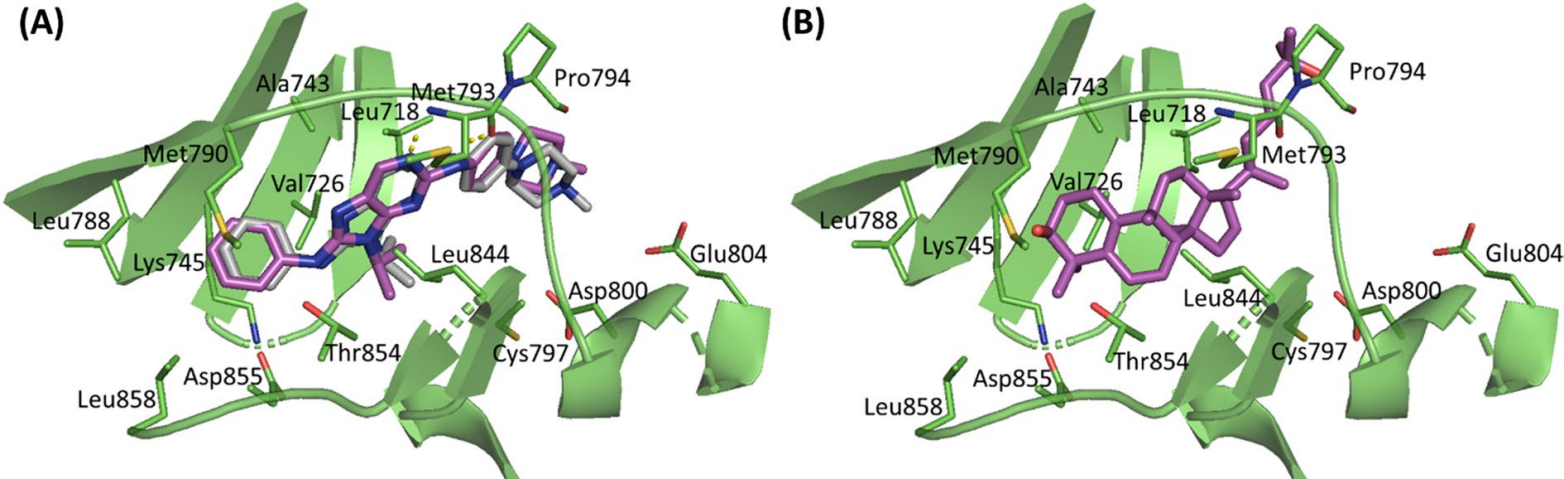
The ATP binding site of EGFR (PDB: 5X2A) (green, cartoon). (**A**) Overlay of crystallized coordinates (grey, sticks) and docked pose (magenta, sticks) of SKLB(3) with a ChemPLP score of 70.94. (**B**) Binding mode of Cucurbita-5(10),6,23-triene-3β,25-diol (magenta, sticks) having a ChemPLP score of 63.31.

EGFR was previously reported to be over-expressed in pancreatic cancer, representing an attractive therapeutic target^17^. Accordingly, we attempted to check the binding mode of the major compound of *C. colocynthis* extract to EGFR. The structure of EGFR complexed with the N8-phenyl-9H-purine-2,8-diamine reversible inhibitor, SKLB(3) (PDB code: 5X2A)^18^, was used to check the binding mode of Cucurbita-5(10),6,23-triene-3β,25-diol. To check the reliability of the resultant docked poses of the docking protocol, the co-crystallized ligand, SKLB (3), was docked into the active site of EGFR. Overlay of the docked pose and the experimentally determined position showed a similar binding mode with a root mean square deviation (RMSD) value of 0.78 Å (Fig. 4A). SKLB (3) occupies the ATP-binding cleft of EGFR, which is mainly lined by hydrophobic residues, with both ends of the aperture lined by polar residues. SKLB (3) completely occupied the aperture, forming several hydrophobic interactions and a few direct and water-mediated H-bonds. Substituents on the N-9 position of purine are sandwiched between Leu718, Val726, and Leu844 sidechains (Fig. 4A). These three residues form what has been previously reported as a "hydrophobic clamp," playing a crucial role in the EGFR binding of reversible inhibitors^18^.

The binding orientation of Cucurbita-5(10),6,23-triene-3β,25-diol is similar to SKLB (3). Its hydrophobic cyclopentaphenanthrene ring, representing the core scaffold of the compound, is sandwiched between the three "hydrophobic clamp" residues (Fig. 4B). The twist in the binding pose of the cyclopentaphenanthrene scaffold makes the cyclopentyl ring mimic the N-9 substituent of SKLB (3), which is believed to be crucial for EGFR tight binding^18^. Additionally, it is anchored from each end with a H-bond interaction between (1) the hydroxyl group of the heptenyl chain and the backbone carbonyl of Pro794 from one end and (2) the 3-hydroxyl group of the cyclopentaphenanthrene ring and the sidechain of Met790 from another end.

**Figure 5:**
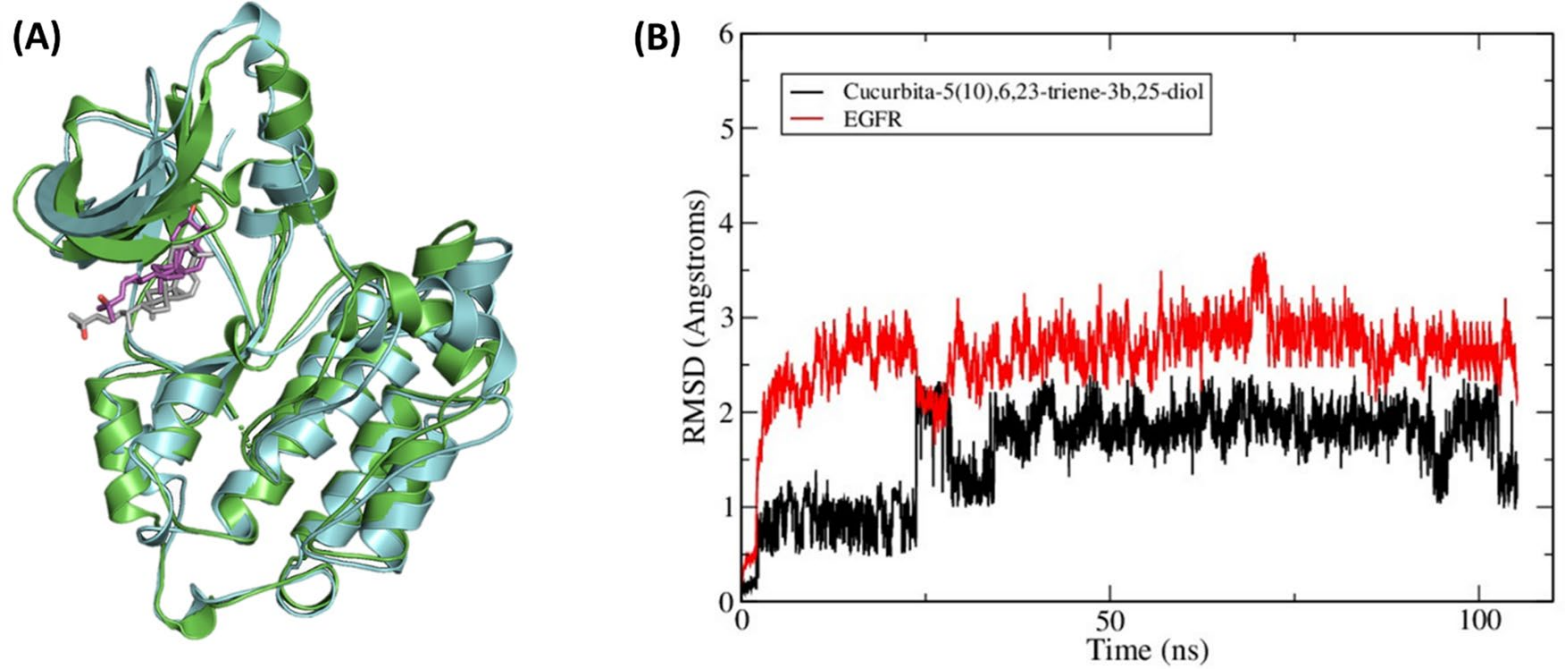
**(A)** Overlay of the initial and a representative frame from the MD simulation for Cucurbita-5(10),6,23-triene-3β,25-diol bound to EGFR. The initial EGFR coordinates are shown as green cartoon with Cucurbita-5(10),6,23-triene-3β,25-diol as grey sticks while the representative MD frame is shown as cyan cartoon with Cucurbita-5(10),6,23-triene-3β,25-diol as magenta sticks (**B**). Plot of root-mean-square deviations (RMSD) of the entire trajectory with frames sampled every 20 ps. The plot depicts RMSD values based on EGFR backbone atoms (red) and ligand heavy atoms (black) between the trajectory frames and the starting geometry.

To confirm the stable binding of Cucurbita-5(10),6,23-triene-3β,25-diol to EGFR, a 100-ns MD simulation for the complex was carried out using AMBER software package^25^. The resultant trajectory was visually examined which showed the retention of Cucurbita-5(10),6,23-triene-3*β*,25-diol within the bidning site of EGFR (Fig. 5A). The initial docking binding mode was confirmed by RMSD analysis of the ligand non-hydrogen atoms which showed little deviation from the initial geometry with a plateau observed at 2 Å after 40 ns (Fig. 5B). Similarly, EGFR maintained its conformation with little deviations observed in the RMSD of the protein’s backbone atoms, with a plateau observed at 2.5 Å after 40 ns. This stable binding mode suggests that the observed anti-pancreatic cancer activity of *C. colocynthis* extract is attributed to EGFR inhibition by Cucurbita-5(10),6,23-triene-3β,25-diol.

## Discussion

Cancer is regarded as one of the most significant public health issues, regardless of the economic standards^1,2^. Medicinal plants are now regarded as a natural source of anticancer medicines due to their antioxidant and anti-mutagenic activities along their low side effects, low cost, and ease of availability. Accordingly, medicinal plants are viewed as an attaractive option for cancer treatment^4^.

This study focused on evaluating the cytotoxic effect of *C.colocynthis* on pancreatic and skin cancer cells. The EtOAc extract of *C. colocynthis* demonstrated significantly favorable effects on cancer cells (PaCa-2 and A-431) while having little to no negative effects on healthy (normal) cells. This cytotoxic selectivity represents a major advantage for the EtOAc extract of *C. colocynthis* over other chemotherapeutic agents that lack this selectivity and kill both cancer and normal cells. Our results appear in agreement with previous studies that showed *C. colocynthis* plant extracts to have cytotoxic effects against some cancer cells^1,2^. Perven et al*.*^12^ reported that the ethanolic fruit extract had cytotoxic effects on the HEp-2 and L929 cancer cell lines in Wistar mice. Alkaloids from fruit extracts had cytotoxic effects against human breast cancer cell lines (MCF-7, HepG-2)^11^. The leaf extracts showed cytotoxic effects and induced apoptosis on the ER-MDAMB-231 and MCF7 cell lines which were used to model human breast cancer^8^.

Chemical analysis of the EtOAc extract of *C. colocynthis* was conducted by comparing the fragmentation patterns in the positive mode to reference compounds' spectra available in literature. Accordingly, a total of 24 compounds were identified with Cucurbita-5(10),6,23-triene-3,25-diol, a derivative of cucurbitacin (a highly oxygenated tetracyclic triterpene) identified as a major component in the extract. Molecular docking experiments showed Cucurbita-5(10), 6,23-triene-3,25-diol to bind effectively in the binding site of EGFR with a 100-ns MD simulation further proving the stability of this complex. The modelling studies strongly suggest Cucurbita-5(10), 6,23-triene-3,25-diol binding to EGFR to be responsible for the observed anticancer activity of the EtOAc extract of *C. colocynthis.* Our results align with previous studies reporting the role of Cucurbita-5(10),6,23-triene-3,25-diol in cancer treatment^34,35^.

In conclusion, the results show a selective cytotoxic effect of the the EtOAc extract of *C. colocynthis* towards pancreatic and skin cancer with Cucurbita-5(10), 6,23-triene-3,25-diol being a major component contributing to this observed effect. This suggests *C. colocynthis* to be a cheap and safe therapeutic to be used in pancreatic and skin cancers.

## Data Availability

The data and materials are available from the corresponding author upon request.
